# ZNF32 protects against oxidative stress-induced apoptosis by modulating C1QBP transcription

**DOI:** 10.18632/oncotarget.5646

**Published:** 2015-10-16

**Authors:** Kai Li, Bo Gao, Jun Li, Haining Chen, Yanyan Li, Yuyan Wei, Di Gong, Junping Gao, Jie Zhang, Weiwei Tan, Tianfu Wen, Le Zhang, Lugang Huang, Rong Xiang, Ping Lin, Yuquan Wei

**Affiliations:** ^1^ Department of Experimental Oncology, State Key Laboratory of Biotherapy, West China Hospital, Sichuan University, and Collaborative Innovation Center for Biotherapy, Chengdu, China; ^2^ Department of Pathology, College of Clinical Medicine, Dali University, Dali, China; ^3^ Department of Gastrointestinal Surgery, West China Hospital, Sichuan University, Chengdu, China; ^4^ Department Biorepository, State Key Laboratory of Biotherapy, West China Hospital, Sichuan University, and Collaborative Innovation Center for Biotherapy, Chengdu, China; ^5^ Department of Liver Surgery, West China Hospital, Sichuan University, Chengdu, China; ^6^ Department of Pediatric Surgery, West China Hospital, Sichuan University, Chengdu, China; ^7^ Department of Clinical Medicine, School of Medicine, Nankai University, and Collaborative Innovation Center for Biotherapy, Tianjin, China; ^8^ Department of Cancer Biotherapy, State Key Laboratory of Biotherapy and Cancer Center, West China Hospital, Sichuan University, and Collaborative Innovation Center for Biotherapy, Chengdu, China

**Keywords:** sp1-ZNF32-C1QBP axis, oxidative stress, transcriptional regulation, mitochondrial membrane potential, pro-oxidant-based anticancer therapy

## Abstract

Reactive oxygen species (ROS)-driven oxidative stress has been recognized as a critical inducer of cancer cell death in response to therapeutic agents. Our previous studies have demonstrated that zinc finger protein (ZNF)32 is key to cell survival upon oxidant stimulation. However, the mechanisms by which ZNF32 mediates cell death remain unclear. Here, we show that at moderate levels of ROS, Sp1 directly binds to two GC boxes within the ZNF32 promoter to activate ZNF32 transcription. Alternatively, at cytotoxic ROS concentrations, ZNF32 expression is repressed due to decreased binding activity of Sp1. ZNF32 overexpression maintains mitochondrial membrane potential and enhances the antioxidant capacity of cells to detoxify ROS, and these effects promote cell survival upon pro-oxidant agent treatment. Alternatively, ZNF32-deficient cells are more sensitive and vulnerable to oxidative stress-induced cell injury. Mechanistically, we demonstrate that complement 1q-binding protein (C1QBP) is a direct target gene of ZNF32 that inactivates the p38 MAPK pathway, thereby exerting the protective effects of ZNF32 on oxidative stress-induced apoptosis. Taken together, our findings indicate a novel mechanism by which the Sp1-ZNF32-C1QBP axis protects against oxidative stress and implicate a promising strategy that ZNF32 inhibition combined with pro-oxidant anticancer agents for hepatocellular carcinoma treatment.

## INTRODUCTION

Apoptosis is a genetically programmed cell death mechanism that is triggered by a variety of extrinsic and intrinsic signals, including various cellular stresses or cytotoxic drugs that may induce the activation of certain pro-apoptotic proteins. Dysfunctional apoptosis has been determined to be a unique trait of cancer development. The mechanism by which cancer cells escape apoptosis remains of great interest in oncological research, and apoptotic signaling has become a major target of cancer treatment. To date, multiple factors, particularly ROS-driven oxidative stress, have been found to induce apoptosis [1, 2].

ROS, including the superoxide radical (O_2_^−^), the hydroxyl radical (HO.), and hydrogen peroxide (H_2_O_2_), are physiologically generated continuously as byproducts of cellular processes such as mitochondrial metabolism. Recent evidence suggests that intracellular biological responses to certain levels of ROS vary greatly [3–5]. At low to moderate levels, ROS act as essential second messengers by which cytokines and growth factors promote cell proliferation and survival [6–8]. Alternatively, excessive ROS levels exert serious oxidative stress on cells, cause damage to cellular components such as DNA, proteins, and lipids, and ultimately induce cell death. ROS has been reported to be involved in various aspects of cancer pathogenesis, including the alteration of cellular signaling, gene expression [9, 10], DNA mutations, genomic stability, [11, 12], and chronic inflammation [13]. Although an increased level of ROS has been observed in cancer cells compared with normal cells, ROS homeostasis can be achieved in cancer cells by elevating antioxidant-scavenging capacity to promote cell proliferation and acquired drug resistance [14–16]. Based on these considerations, ROS homeostasis has been demonstrated to be disturbed via the suppression of the antioxidant system or the direct addition of ROS inducers; these therapies synergistically enhance the cytotoxicity of pro-oxidant-based anticancer agents and promote cancer cell death. These findings implicate ROS homeostasis as a potential target for cancer treatment [17–19].

Mitochondria perform critical functions in many cellular processes, including ATP production, fatty acid oxidation, apoptosis, and necrosis [20–22]. Mitochondria represent both the primary source of ROS and the primary target of ROS damage. Excess ROS generated by mitochondria cause mitochondrial permeability transition pore (mPTP) opening, which results in the loss of mitochondrial membrane potential; increased ROS production; mitochondrial rupture and dysfunction; and, ultimately, cell death [23–25]. C1QBP, a conserved eukaryotic protein that is predominantly localized to mitochondria, is an essential component of the mPTP complex that contributes to mitochondrial-dependent cell death [26]. Notably, the mPTP, which has been shown to be a causative factor in the pathogenesis of many diseases, is another target for anti-cancer therapy [27–29]. For example, 2-methoxyestradiol has been shown to inhibit the proliferation of and induce apoptosis in human neuroblastoma cells by increasing the ROS levels and abolishing the mitochondrial membrane potential [30].

Zinc finger protein (ZNF)32 is a newly identified ZNF in Homo sapiens that belongs to the Krüppel-like family of transcription factors (KLF). In our previous study, we demonstrated that overexpression of the mouse ZNF32 homolog Zfp637 markedly increased mTERT expression and telomerase activity, maintained telomere length, and inhibited both H_2_O_2_- and D-galactose-induced cell death, which is accompanied by reduced ROS production [31]. Moreover, ZNF32 inhibits autophagy initiation by activating the AKT/mTOR pathway to protect breast cancer cells from stimulus-induced cell death [32]. However, how ZNF32 transcription is modulated during oxidative stress and the mechanisms by which ZNF32 influences cell viability in response to oxidative stress remain unknown.

In this study, we show that the expression of ZNF32 is regulated by Sp1-mediated transcription in response to oxidative stress. ZNF32 downregulation promotes the apoptosis of hepatocellular carcinoma (HCC) cells exposed to the pro-oxidant agents, whereas ZNF32 overexpression protects against this effect. C1QBP is recognized as a target gene of ZNF32 that is essential for ZNF32 to sustain mitochondrial membrane potential and enhance cellular resistance to oxidative stress. These findings demonstrate that the Sp1-ZNF32-C1QBP axis resists oxidative stress and implicate ZNF32 as a potential target to enhance the efficacy of pro-oxidant agents in cancer therapy.

## RESULTS

### ZNF32 is regulated by Sp1 at the transcriptional level in response to oxidative stress

Previously, we showed that the expression of mouse ZNF32 homolog Zfp637 was significantly altered in NIH3T3 fibroblasts when treated with H_2_O_2_, indicating a potential role of ZNF32 in oxidative stress [33]. To determine whether oxidative stress modulates ZNF32 expression in humans, HepG2 cells were treated with H_2_O_2_ in a dose- and time-dependent manner. Interestingly, the expression of ZNF32 increased following low-dose H_2_O_2_ treatment (0.1 or 0.25 mM) for 24 h but decreased gradually when treated with higher doses of H_2_O_2_ (greater than 0.5 mM) (Supplementary Figure S1A). Similarly, the expression of ZNF32 increased in response to acute exposure to 0.5 mM H_2_O_2_ for up to 2 h and then declined by 4 h (Supplementary Figure S1B). To further clarity whether these responses were redox-specific, HepG2 cells were pretreated with the antioxidant agent N-acetyl-L-cysteine (NAC) or butylated hydroxyanisole (BHA). Pretreatment with either NAC or BHA significantly ameliorated the decrease in ZNF32 expression induced by treatment with 0.5 mM H_2_O_2_ for 24 h as well as the increase in ZNF32 expression induced by treatment with 0.1 mM H_2_O_2_ for 24 h or with 0.75 mM H_2_O_2_ for 1 h (Supplementary Figures S1C–S1E).

**Figure 1:**
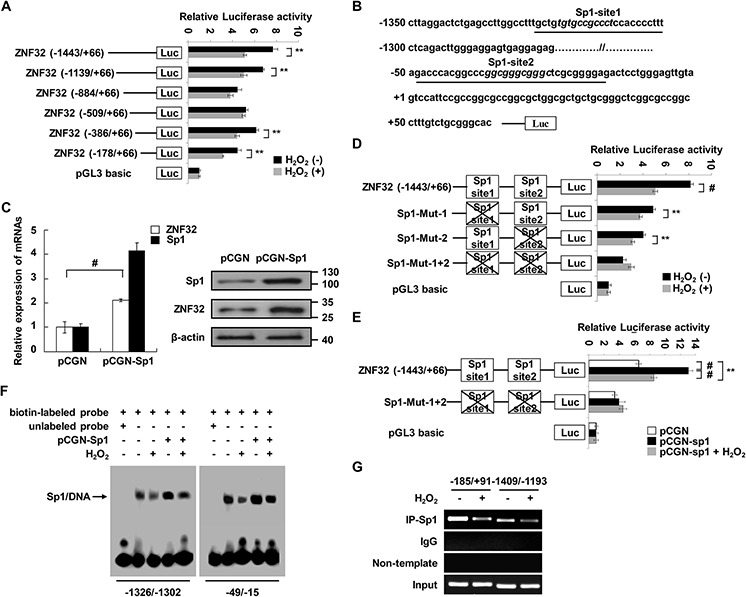
Sp1 transcriptionally regulates ZNF32 expression in response to oxidative stress **A.** HEK293 cells were transiently transfected with ZNF32 promoter 5′-deletion mutant constructs, treated with 0.5 mM H_2_O_2_ for 24 h and then analyzed using a dual luciferase reporter assay. **B.** Schematic representation of the two Sp1-binding sites in the ZNF32 promoter. The consensus sequences for the predicted Sp1 sites (GC boxes) are shown in italics; the oligonucleotides used in EMSA are underlined; and the transcription start site is indicated by +1. **C.** HEK293 cells were transiently transfected with the indicated constructs and then subjected to qRT-PCR and immunoblot analysis. **D.** and **E.** HEK293 cells were transiently transfected with the indicated constructs, treated as in (A) and then analyzed using a dual luciferase reporter assay. **F.** Nuclear extracts from the indicated cells were incubated in biotin-labeled oligonucleotides corresponding to the ZNF32 promoter region (−1326/−1302 bp) or (−49/−15 bp). The arrow shows the specific DNA-protein complex. **G.** DNA fragments from HEK293 cells treated as in (A) were immunoprecipitated with Sp1-specific antibodies and analyzed via RT-PCR using the indicated primers. The data are presented as the mean values ± SEM. Each experiment was performed at least in triplicate, producing consistent results. *p < 0.05, **p < 0.01, #*p* < 0.001.

To identify potential transcriptional regulators of ZNF32 under oxidative stress conditions, functional 5′-deletion analysis of an approximately 1.5-kb region of the ZNF32 promoter was performed by generating a series of deletion mutants fused to a luciferase reporter gene. As shown in Figure 1A, the activities of the (−1443/+66), (−1139/+66), (−386/+66) and (−178/+66) regions of the ZNF32 promoter were significantly decreased by 0.5 mM H_2_O_2_ treatment. Next, we analyzed the ZNF32 promoter sequence and found two putative Sp1 response elements, located at −1318/−1304 and −43/−27; each of which was within an oxidative stress-responsive region (Figure 1B). The transcription factor Sp1 has been shown to mediate oxidative stress–induced gene transcription [34–36]. Thus, we sought to determine whether ZNF32 expression is regulated by Sp1. As shown in Figure 1C, higher mRNA and protein levels of ZNF32 were observed in Sp1-overexpressing cells than in control cells. To locate the Sp1-binding sites, we sequentially engineered luciferase reporter constructs that were mutated at one or two sites from the corresponding wild-type construct. As shown in Figure 1D, compared with wild-type ZNF32 (−1443/+66), functional loss of either of the identified Sp1-response elements inhibited basal luciferase activity at the ZNF32 promoter. Moreover, H_2_O_2_ treatment significantly decreased the transcriptional activity of the wild-type and each single Sp1 site-mutated ZNF32 promoter but not the mutant ZNF32 promoters in which both Sp1 sites were disrupted (Figure 1D). In a parallel experiment, H_2_O_2_ attenuated the increase in transcription at the ZNF32 promoter via Sp1 overexpression (Figure 1E). Electrophoretic mobility shift assay (EMSA) revealed that nuclear extracts from HEK293 cells interacted with each probe containing an Sp1 site derived from the ZNF32 promoter but that addition of 0.5 mM H_2_O_2_ decreased the levels of the shifted complex (Figure 1F). Moreover, overexpression of Sp1 increased the formation of DNA-nucleoprotein complexes, but this effect was attenuated by 0.5 mM H_2_O_2_ treatment (Figure 1F). Accordingly, a chromatin immunoprecipitation (ChIP) assay showed decreased binding of Sp1 to the endogenous ZNF32 promoter regions containing GC-rich boxes after treatment with 0.5 mM H_2_O_2_ (Figure 1G).

As ZNF32 transcription was increased upon treatment with a low dose of H_2_O_2_, we investigated whether the expression of ZNF32 was also induced by Sp1 in response to 0.1 mM H_2_O_2_. As shown in Supplementary Figure S1F, 0.1 mM H_2_O_2_ treatment elevated the luciferase activity of ZNF32 (−1443/+66) but not the Sp1 site double-mutant ZNF32 promoter. Notably, 0.1 mM H_2_O_2_ and Sp1 synergistically facilitated ZNF32 transcription (Supplementary Figure S1F). Consistently, the binding activity of Sp1 was augmented by 0.1 mM H_2_O_2_ treatment (Supplementary Figure S1G). Taken together, these results suggest that Sp1 delicately regulates the transcription of ZNF32 upon oxidative stress, clearly implying a function of ZNF32 in oxidative stress.

### Overexpression of ZNF32 increases the threshold for oxidative stress-induced apoptosis

**Figure 2:**
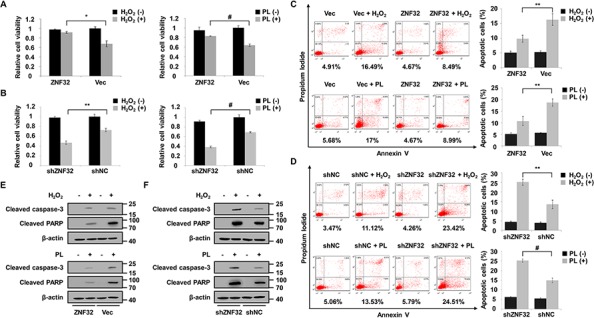
ZNF32 protects against oxidative stress-induced apoptosis **A.** HepG2 cells stably expressing ZNF32 (labeled ZNF32) or control (labeled Vec) were treated with 0.5 mM H_2_O_2_ or 10 μM PL for 24 h and analyzed for cell viability by an MTT assay. **B.** Cell viability analysis in stably ZNF32-silenced (termed shZNF32) or control (termed shNC) HepG2 cells were treated as in (A) for 24 h. **C.** and **D.** Annexin V analysis of apoptosis in the indicated HepG2 cells treated as in (A) **E.** and **F.** The indicated HepG2 cells were treated as in (A) and then subjected to immunoblot analysis using the indicated antibodies. The data are represented as the means ± SEM. Each experiment was performed at least in triplicate, producing consistent results. *p < 0.05, **p < 0.01, #*p* < 0.001.

To address whether ZNF32 plays a functional role in oxidative stress-induced apoptosis, we altered the ZNF32 levels in HepG2 cells via ectopic expression of ZNF32 cDNA or ZNF32-specific short hairpin RNA (shRNA) using lentiviral technology (Supplementary Figure S2). Cells were treated with H_2_O_2_ or piperlongumine (PL), a pro-oxidant drug that has been well characterized to selectively kill cancer cells [37, 38]. We found that neither overexpression nor depletion of ZNF32 significantly affected the viability of HepG2 cells (Figures 2A and 2B). However, when the cells were treated with H_2_O_2_ or PL, increased cell viability was observed in ZNF32-overexpressing cells compared with control cells (Figure 2A), whereas ZNF32-silenced cells exhibited significantly decreased cell viability (Figure 2B). These findings were confirmed by an Annexin V/propidium iodide (PI) assay (Figures 2C and 2D). Furthermore, when the cells were treated with H_2_O_2_ or PL, the levels of two apoptosis markers, cleaved caspase 3 and cleaved poly(ADP-ribose) polymerase (PARP), were significantly increased in ZNF32-knockdown cells, but this pro-apoptotic effect was largely reversed by ZNF32 overexpression (Figures 2E and 2F). Taken together, these results suggest that ZNF32 overexpression protects against oxidative stress-induced cell injury but that knockdown of ZNF32 renders cells more sensitive and vulnerable to oxidative challenge. These findings implicate ZNF32 as a negative regulator of oxidative stress-induced apoptosis.

### Overexpression of ZNF32 protects mitochondrial membrane potential and the antioxidant system upon oxidative stress

**Figure 3:**
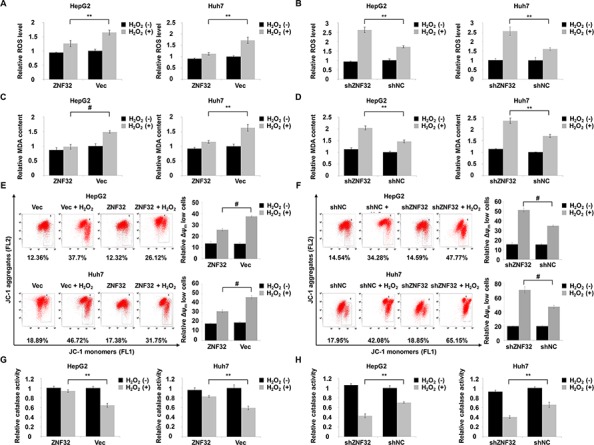
ZNF32 contributes to ROS defense and the maintenance of mitochondrial membrane potential in response to oxidative stress **A.** HepG2 and Huh7 cells stably expressing ZNF32 and incubated in 0.5 mM H_2_O_2_ for 24 h were stained with DCFH-DA. DCF fluorescence intensity was determined using a fluorescence microplate reader. **B.** The indicated cells treated as in (A) were stained with DCFH-DA and subjected to fluorescence intensity analysis to determine the intracellular ROS levels. **C.** and **D.** The indicated cells were treated as in (A) and assessed for the lipid peroxidation level using the MDA assay. **E.** and **F.** The indicated cells were treated as in (A) and subjected to measurement of the mitochondrial membrane potential using the JC-1 assay. **G.** and **H.** The indicated cells treated as in (A) were subjected to catalase activity analysis to determine the intracellular antioxidant capacity. The data are presented as the mean values ± SEM. Each experiment was performed at least in triplicate, producing consistent results. *p < 0.05, **p < 0.01.

Next, we investigated the mechanisms underlying the ZNF32-dependent modulation of H_2_O_2_ damage. ZNF32-overexpressing cells showed a lower ROS level than control cells following the exogenous addition of H_2_O_2_ (Figure 3A). Conversely, the intracellular concentrations of ROS were significantly increased in ZNF32-depleted cells treated with H_2_O_2_ (Figure 3B). Next, we measured the levels of malondialdehyde (MDA), a marker of lipid peroxidation damage, in cell culture supernatants. Similar to the above results, a protective effect of ZNF32 overexpression was observed in cells exposed to H_2_O_2_ (Figure 3C), but oxidative stress-induced MDA formation was significantly increased in ZNF32-silenced cells (Figure 3D). Mitochondria perform critical functions in oxidative stress-induced apoptosis, and one mechanism by which mitochondrial failure causes apoptosis involves the loss of mitochondrial membrane potential and the generation of ROS [39]. As ZNF32 overexpression suppressed the ROS production and apoptosis induced by H_2_O_2_, we further tested whether ZNF32 affects the mitochondrial membrane potential under oxidative stress. Consistently, ZNF32 overexpression greatly suppressed oxidative stress-induced mPTP opening according to the fluorescent JC-1 aggregation assay (Figure 3E), whereas knockdown of ZNF32 greatly reduced the mitochondrial membrane potential upon H_2_O_2_ treatment (Figure 3F). To further clarity whether ZNF32 activates intracellular antioxidant systems, we detected the activity of catalase, which converts H_2_O_2_ into O_2_ and H_2_O. In agreement with the above results, ZNF32 overexpression cells showed higher catalase activity than control cells following H_2_O_2_ treatment (Figure 3G). In contrast, catalase activity was significantly decreased in ZNF32-depleted cells following H_2_O_2_ treatment (Figure 3H). Similar phenomena were observed in HepG2 cells treated with PL (Supplementary Figure S3). In summary, our results implied that ZNF32-deficient cells were more sensitive to oxidative stress-induced apoptosis due to mitochondrial dysfunction and antioxidant system deficiency.

### C1QBP is essential for ZNF32 to sustain the mitochondrial membrane potential during oxidative stress

**Figure 4:**
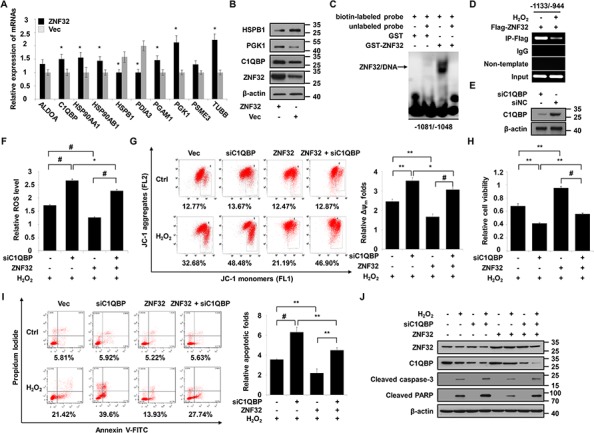
C1QBP is a target gene of ZNF32 that is required for the protective effects of ZNF32 on oxidative stress-induced apoptosis **A.** RNA isolated from HepG2 cells stably expressing ZNF32 or from control cells was probed via qRT-PCR using the indicated primers. **B.** The indicated cells were subjected to immunoblot analysis using the indicated antibodies. **C.** EMSA was performed using the GST-ZNF32 fusion protein and biotin-labeled oligonucleotides from the C1QBP promoter region (−1081/−1048 bp) in the presence or absence of unlabeled double-stranded probes. The arrows indicate the specific complexes. **D.** DNA fragments from HEK293 cells transfected with Flag-ZNF32 were treated with or without 0.5 mM H_2_O_2_, immunoprecipitated with Flag-specific antibodies, and analyzed via RT-PCR using the indicated primers. **E.** ZNF32-overexpressing HepG2 cells were transiently transfected with the indicated siRNAs and then analyzed by immunoblot using the indicated antibodies. **F.** The indicated HepG2 cells were transiently transfected with C1QBP siRNA, treated with 0.5 mM H_2_O_2_ for 24 h, stained with DCFH-DA and then subjected to fluorescence intensity analysis to determine the intracellular ROS levels. **G.** The indicated HepG2 cells were transfected, treated as in (F), and then assayed for the mitochondrial membrane potential using the JC-1 assay. **H.** The indicated HepG2 cells were transfected, treated as in (F), and then analyzed for cell viability. **I.** The indicated HepG2 cells were transfected, treated as in (F) and then subjected to Annexin V analysis. **J.** The indicated HepG2 cells were transfected, treated as in (F) and then subjected to immunoblot analysis using the indicated antibodies. The data are presented as the mean values ± SEM. Each experiment was performed at least in triplicate, producing consistent results. *p < 0.05, **p < 0.01, #*p* < 0.001.

To further elucidate the molecular mechanisms underlying the decreased susceptibility of ZNF32-overexpressing cells to oxidative stress, two-dimensional polyacrylamide gel electrophoresis (2-DE)-based proteomic analysis was performed to identify the key responsive proteins involved in this process. Representative paired gel images from the 2-DE analysis are shown in Supplementary Figure S4A. Detailed information for the 24 identified proteins is provided in Table 1. The identified proteins were divided into various groups based on their subcellular localization and biological functions (Supplementary Figures S4B and S4C). The intrinsic interactions of these proteins were analyzed using the web-based tool STRING. The apoptosis-regulating proteins and stress-responsive proteins were grouped into specific clusters (Supplementary Figure S4D). Interestingly, these proteins were linked together when MAPK1 (also known as p38) and TNFα, two critical proteins involved in ROS and cell death signaling, were added to the analysis (Supplementary Figure S4E). Among the 24 identified proteins, we selected 10 genes that were associated with cell proliferation, metabolism and stress responses to validate their correlation with ZNF32. As shown in Figure 4A, 8 of the 10 selected genes were significantly correlated with ZNF32. Next, 3 of these 10 genes were chosen for further examination by immunoblot analysis. Consistently, ZNF32 overexpression induced elevated C1QBP and PGK1 protein expression but repressed HSPB1 expression (Figure 4B). Given the critical role of mitochondria in the regulation of ROS homeostasis and apoptosis, we inferred that the mechanisms by which ZNF32 suppression caused apoptosis upon oxidant treatment involved the loss of mitochondrial membrane potential, followed by increased ROS generation, mitochondrial dysfunction and, ultimately, caspase cascade activation. C1QBP, a substantial component of the mPTP complex, is involved in regulating oxidative phosphorylation and inner mitochondrial membrane permeability [40]. As aberrant regulation of C1QBP is sufficient to induce mPTP opening and subsequent mitochondria-dependent apoptosis [41–43], C1QBP was selected for further mechanistic study.

**Table 1:** Protein spots identified by ESI-Q-TOF MS

Protein Spot No.	Protein Name	Accession No.	Protein Score	Protein MW	Protein PI	Peptide Count	AA Coverage %
1	PGK1	IPI00169383	220	44615	8.3	13	49
2	ALDOA	IPI00796333	262	45261	8.48	16	51
3	GAPDH	IPI00789134	146	27853	6.45	4	35
4	PGAM1	IPI00453476	54	28850	6.67	3	20
5	DTNA	IPI00218655	46	22094	6.46	7	5
6	PDIA3	IPI00025252	69	56747	5.98	8	13
7	CAPN9	IPI00016919	36	76120	5.45	5	31
8	ANXA6	IPI00221226	17	75894	5.42	1	1
9	ENO3	IPI00909595	47	29508	5.46	3	13
10	HSPB1	IPI00025512	130	22783	5.98	9	57
11	PSME3	IPI22019445	49	30887	5.79	3	16
12	CATSPERG	IPI00917065	34	33059	5.51	5	15
13	HNRNPK	IPI00910458	58	41781	5.43	4	11
14	HSP90AB1	IPI00414676	190	83265	4.97	11	22
15	HSP90AA1	IPI00382470	358	98162	5.07	21	29
16	HNRNPA2B1	IPI00916517	141	28394	4.79	8	17
17	IL18	IPI00980067	72	21882	4.66	6	27
18	LUC7L2	IPI00893315	40	9431.6	4.93	4	4
19	TUBB	IPI00011654	343	49671	4.78	19	67
20	C1QBP	IPI00014230	78	31362	4.74	4	16
21	HNRNPC	IPI00759596	116	27805	4.55	6	26
22	CALM1	IPI00916600	84	21675	4.45	3	16
23	PTGES3	IPI00789101	67	19449	4.76	4	27
24	TPRG1	IPI00926817	31	11235	4.56	3	7

To explore whether C1QBP is involved in resistance to oxidative stress, we first determined the expression of C1QBP upon treatment with increasing concentrations of H_2_O_2_. Our data showed that lower H_2_O_2_ levels (0.1 and 0.25 mM) increased C1QBP expression but that higher levels of H_2_O_2_ (0.5, 0.75 and 1 mM) treatment inhibited C1QBP transcription (Supplementary Figure S5A). Moreover, C1QBP knockdown promoted apoptosis and reduced the viability of HepG2 cells treated with 0.5 mM H_2_O_2_ for 24 h compared with endogenous C1QBP expression. However, no significant alterations were observed in C1QBP-silenced HepG2 cells treated with 0.1 mM or 0.25 mM H_2_O_2_ for 24 h (Supplementary Figures S5B and S5C). These results indicate that C1QBP protects cells from oxidative stress-induced apoptosis.

Based on the structure and localization of ZNF32, we inferred that ZNF32 may function as a transcription factor and identified a consensus binding site in ZNF32 (GACTTT) based on cyclic amplification and selection of targets (manuscript in preparation). The C1QBP promoter sequence was analyzed, and one putative ZNF32-binding site, located at −1067/−1062 bp, was found. We confirmed that the GST-ZNF32 fusion protein formed protein-DNA complexes with oligonucleotides derived from the C1QBP promoter (−1081/−1048 bp) (Figure 4C). In agreement with the above results, the ChIP assay revealed that ZNF32 directly binds to the promoter of C1QBP but that this binding is decreased by H_2_O_2_ treatment (Figure 4D). To address the role of C1QBP in the ZNF32-dependent regulation of apoptosis in response to oxidative stress, we depleted C1QBP using specific siRNA in ZNF32-overexpressing HepG2 cells (Figure 4E). C1QBP-deficient cells exhibited increased ROS generation and reduced mitochondrial membrane potential upon H_2_O_2_ treatment despite ZNF32 overexpression (Figures 4F and 4G). Accordingly, the protective effect of ZNF32 overexpression against H_2_O_2_-induced toxicity was reversed by the depletion of C1QBP (Figures 4H–4J). These results indicate that the ZNF32-C1QBP signaling axis regulates cell susceptibility to oxidative stress-induced apoptosis.

### C1QBP knockdown facilitates the activation of the p38 MAPK pathway in response to oxidative stress

**Figure 5:**
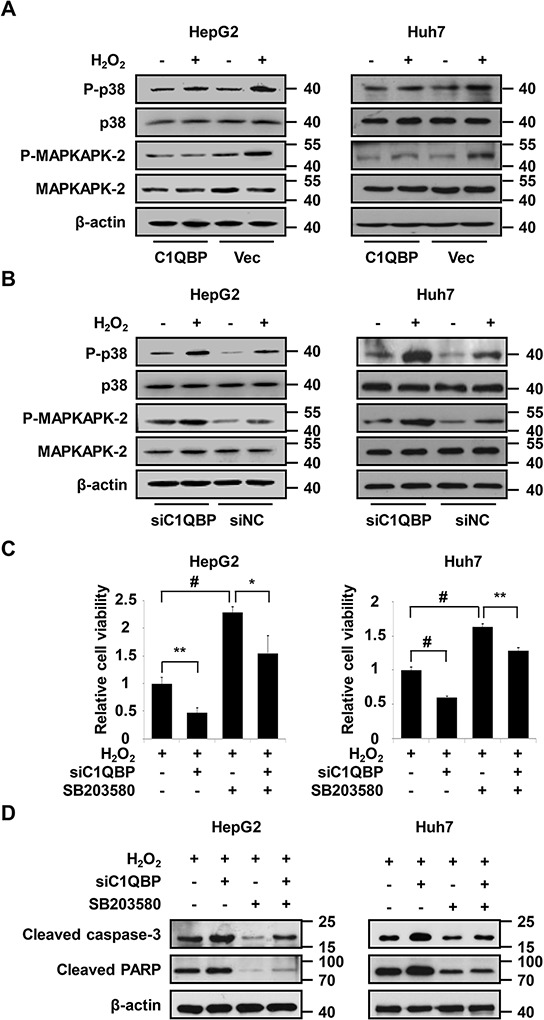
Activation of p38 MAPK is essential for C1QBP deficiency-mediated apoptosis under oxidative stress **A.** The indicated cells were transfected with C1QBP expression plasmid or vector, treated with 0.5 mM H_2_O_2_ for 24 h and then subjected to immunoblot analysis using the indicated antibodies. **B.** The indicated cells were transfected with C1QBP siRNA or siNC, treated as in (A) and then subjected to immunoblot analysis using the indicated antibodies. **C.** The indicated cells were transfected with C1QBP siRNA or siNC, pretreated with SB203580 for 1 h, treated with 0.5 mM H_2_O_2_ for 24 h, and then subjected to cell viability analysis. **D.** The indicated cells were transfected with C1QBP siRNA or siNC, treated as in (C) and then subjected to immunoblot analysis using the indicated antibodies. The data are presented as the mean values ± SEM. Each experiment was performed at least in triplicate, producing consistent results. *p < 0.05, **p < 0.01, #*p* < 0.001.

As described above, C1QBP knockdown accelerates ROS accumulation upon oxidant treatment, and STRING network analysis demonstrates a link between C1QBP and p38 MAPK (Figure 4F and Supplementary Figure S4E). Moreover, a previous study demonstrated that p38 MAPK acts as a sensor of ROS during tumorigenesis and apoptosis [44]. To investigate whether C1QBP influences p38 MAPK pathway activation upon H_2_O_2_ treatment, the phosphorylation of p38 MAPK and its downstream target MAPKAPK-2 was measured by western blot. As shown in Figure 5A, overexpression of C1QBP inhibited the H_2_O_2_-induced phosphorylation of p38 MAPK and MAPKAPK-2 compared with endogenous C1QBP expression. Conversely, H_2_O_2_ further stimulated the p38 MAPK pathway in C1QBP-silenced cells (Figure 5B). To determine whether C1QBP functions in apoptosis via a p38 MAPK-dependent pathway, we pretreated cells with the p38 MAPK inhibitor SB203580 prior to H_2_O_2_ treatment. Interestingly, the inhibition of p38 MAPK significantly attenuated the decrease the viability of C1QBP-silenced cells treated with H_2_O_2_ (Figure 5C). Consistently, SB203580 treatment effectively suppressed the formation of cleaved caspase 3 and cleaved PARP in C1QBP-silenced cells following H_2_O_2_ treatment (Figure 5D). Similar phenomena were observed in HepG2 cells treated with PL (Supplementary Figures S6A–S6C). To further determine the influence of ZNF32 on the activation of the p38 MAPK signaling pathway, ZNF32-overexpressing HepG2 cells were pretreated with SB203580, followed by treatment with 0.5 mM H_2_O_2_ for 24 h. Interestingly, overexpression of ZNF32 combined with SB203580 pretreatment resulted in reduced apoptosis and increased cell viability compared with ZNF32 overexpression or SB203580 treatment alone (Supplementary Figures S6D and S6E). Taken together, our results show that oxidative stress further activates the p38 MAPK signaling pathway in ZNF32- and C1QBP-deficient cells and suggest that the ZNF32-C1QBP axis participates in the regulation of oxidative stress-induced apoptosis at least partially via the p38 MAPK pathway.

### ZNF32 deficiency augments the anti-tumor effect of the pro-oxidant drug PL in mice

**Figure 6:**
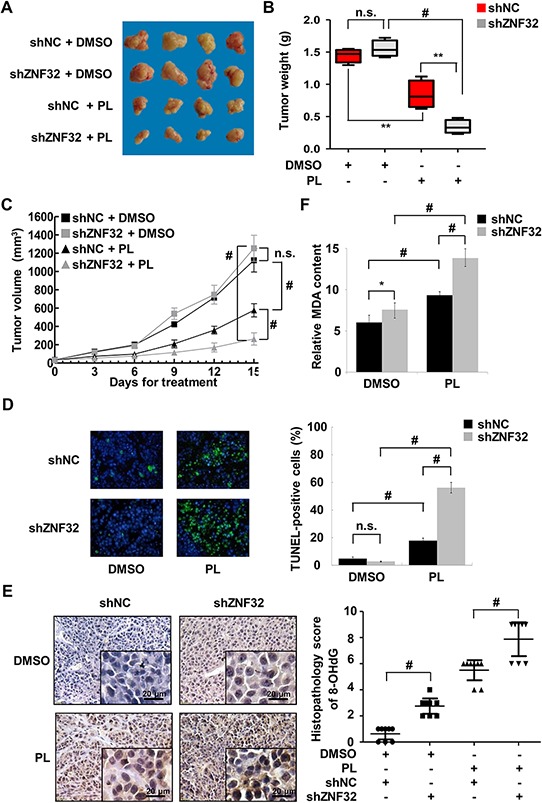
ZNF32 knockdown enhances the toxicity of the pro-oxidant drug PL *in vivo* **A.** Mice were injected with HepG2 cells stably expressing shZNF32 or shNC. When the diameter of tumor xenografts reached approximately 3 mm, the mice were injected daily with PL (2.5 mg/kg) or DMSO. Mice were euthanized 15 days after treatment. Representative images of the tumors are shown. **B.** In the box plots, the final tumor weights are presented (*n* = 8 per group). The whiskers in the box plots represent the maximum and minimum values. **C.** Tumor volume analysis of xenografts of the indicated cells (*n* = 8 per group). **D.** Tumor xenograft tissues were fixed with 4% paraformaldehyde, processed, embedded in paraffin wax and then assessed for cellular apoptosis using a TUNEL assay. **E.** Oxidative DNA damage in tumor xenograft tissues was detected by quantifying 8-OHdG expression. **F.** Tumor xenograft tissues were homogenized and then assessed for the lipid peroxidation levels using an MDA assay. The data are presented as the mean values ± SEM. Each experiment was performed at least in triplicate, producing consistent results. *p < 0.05, **p < 0.01, #*p* < 0.001.

As the results presented above suggested that ZNF32 regulates cell survival upon oxidative stress *in vitro*, we investigated whether ZNF32 influences the effect of the pro-oxidant drug PL on HepG2 cells *in vivo*. Knockdown of ZNF32 slightly accelerated tumor xenograft growth in DMSO-treated control mice (Figures 6A–6C). However, PL treatment caused a remarkable reduction in tumor weight and volume for xenografts derived from ZNF32-silenced cells compared to those from control cells. These findings were confirmed by a terminal deoxynucleotidyl transferase-mediated dUTP nick end labeling (TUNEL) assay (Figure 6D). Next, to determine whether ZNF32 reduces oxidative damage *in vivo*, the content of 8-hydroxydeoxyguanosine (8-OHdG), a marker of oxidative DNA damage, was determined in the sections of tumor tissue from xenografted animals. Intriguingly, immunohistochemistry revealed elevated levels of 8-OHdG in ZNF32-silenced cell-based xenografts (Figure 6E). Moreover, PL treatment resulted in increased oxidative damage in ZNF32-silenced cell-based xenografts compared with control cell-based xenografts (Figure 6E). Similar results were observed for MDA formation in xenograft tumors of mice treated with PL (Figure 6F). Overall, these data suggest that ZNF32 expression confers resistance to pro-oxidant drugs *in vivo*.

### ZNF32 expression is positively correlated with C1QBP expression and is associated with necrosis and histodifferentiation in HCC samples

**Figure 7:**
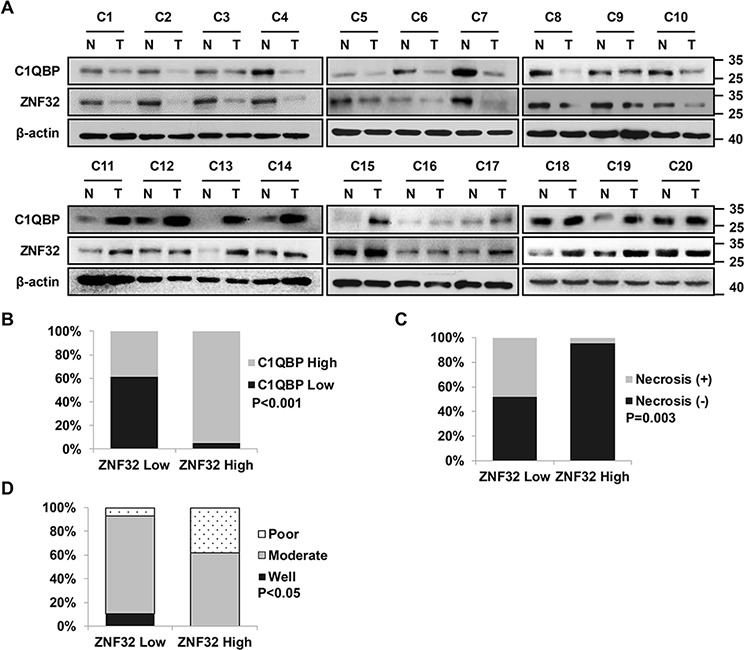
Correlation analysis of ZNF32 expression in human HCC samples **A.** Clinical hepatocellular carcinoma (HCC) cases displaying decreased ZNF32 and C1QBP protein levels. Human HCC samples paired with carcinoma tissue (labeled as T) and adjacent normal tissue (labeled as N) were lysed and then subjected to immunoblot analysis using the indicated antibodies. **B.** Correlation analysis of ZNF32 and C1QBP protein expression in HCC samples. **C.** Correlation analysis of ZNF32 expression and necrosis in HCC samples. **D.** Correlation analysis of ZNF32 expression and histodifferentiation in HCC samples. *p* values were calculated using Fisher's exact test.

We next examined the expression of ZNF32 and its downstream target C1QBP in HCC samples. A total of 50 paired primary human HCC and adjacent normal hepatic tissue samples were collected. The clinicopathological characteristics of the patients are summarized in Table 2. Compared to the matched normal hepatic tissues, the HCC tissues displayed a significant decrease in ZNF32 and C1QBP expression in 29 and 20 of the cases, respectively. Notably, co-expression of ZNF32 and C1QBP was observed in 39 of the 50 paired samples (78%), including co-upregulation in 20 paired samples and co-downregulation in 19 paired samples (Figures 7A and 7B); these results suggest that ZNF32 expression is positively correlated with C1QBP expression. Moreover, our data indicated no apparent association between ZNF32 expression and age, sex, stage, HBV infection, or fibrosis. Intriguingly, the expression of ZNF32 was lower in 14 out of the 15 HCC samples exhibiting necrosis than in the matched normal hepatic tissue samples (*p* = 0.003, Figure 7C). In addition, increased expression of ZNF32 significantly correlated with poor differentiation (*p* = 0.012, Figure 7D).

**Table 2:** Clinicopathological characteristics of the 50 analyzed hepatocellular carcinoma patients

	Low ZNF32 Expression	High ZNF32 Expression	*p* value
Age (years)			
< 55	19	9	0.11
≥ 55	10	12	
Sex			
Male	27	21	0.50
Female	2	0	
TNM stage			
I	10	9	0.93
II	9	5	
III	8	6	
IV	2	1	
Histodifferentiation			
High differentiation	3	0	0.012
Moderate differentiation	24	13	
Poor differentiation	2	8	
HBV status			
Positive	20	16	0.57
Negative	9	5	
Necrosis			
Yes	14	1	0.003
No	15	20	
Fibrosis			
F0	9	7	0.74
F1	20	14	

## DISCUSSION

The regulation of redox homeostasis is fundamental to maintaining normal cellular functions and promoting cell survival. Accompanied with a higher ROS level than normal cells, cancer cells characteristically develop several adaptive responses to maintain ROS levels that are compatible with cellular biological functions. Thus, interferences in ROS homeostasis are believed to be capable of disrupting cancer cell biological metabolism and efficiently inducing cell death [17, 18]. For example, PGC1α reduces the generation of mitochondrial-driven ROS to promote survival under oxidative stress conditions, and the pro-oxidant drugs PL and PEITC show markedly increased potency in PGC1α-deficient melanoma cells [45]. In the present study, we report that ZNF32 suppresses ROS accumulation and MDA formation and rescues mitochondrial membrane potential and catalase activity to enable cell survival under oxidative stress. Conversely, ZNF32-deficient cells are more sensitive and vulnerable to oxidative stress. In tumor xenografts, knockdown of ZNF32 markedly increased the potency of the pro-oxidant drug PL (Figure 6), leading to enhanced tumor suppression.

KLF includes a set of zinc finger DNA-binding proteins that are involved in cell proliferation and apoptosis via the regulation of gene expression [46, 47]. ZNF32 was recently identified as a novel KLF, and its downstream targets have rarely been reported in the literature. Here, we demonstrate that ZNF32 regulates C1QBP expression by directly binding to the C1QBP promoter, where the transcriptional activity of ZNF32 is suppressed by toxic doses of H_2_O_2_ (Figures 4C and 4D). Moreover, depletion of C1QBP reverses the protective effect of ZNF32 against H_2_O_2_-induced mitochondrial dysfunction; this finding suggests that C1QBP is essential for ZNF32 to sustain the mitochondrial membrane potential and ROS homeostasis and to resist apoptosis in response to oxidative stress (Figures 4F–4J). In line with our findings, McGee and his colleague have reported that neither overexpression nor knockdown of C1QBP alters mitochondrial function at baseline. However, overexpression of C1QBP in mitochondria greatly suppresses oxidative stress-induced mPTP opening and leads to mitochondrial dysfunction, whereas depletion of C1QBP exerts the opposite effect and instead sensitizes cells to H_2_O_2_-induced cytotoxicity and cell death [40]. Further support for these conclusions is based on the observation that the loss of C1QBP does not significantly decrease cell viability but does negatively impact the survival of cells treated with cisplatin, a well-documented pro-oxidant agent [48]. Thus, our data suggest that ZNF32 acts as a stress-responsive factor to control intracellular ROS accumulation and cell susceptibility to oxidative stress via the regulation of C1QBP transcription and mitochondrial membrane potential.

Despite the essential role of ZNF32 in ROS homeostasis, it is necessary to understand the mechanism by which ZNF32 is regulated in response to oxidative stress. Here, we demonstrate that Sp1 specifically and directly binds to two GC boxes within the ZNF32 promoter and that the binding activity of Sp1 is regulated by different concentrations of H_2_O_2_ (Figure 1 and Supplementary Figure S1). Sp1 is a redox-regulated transcription factor that can act as an anti-death transcription factor by regulating the expression of various target genes, such as the Kv1.5 potassium channel gene and insulin receptor substrate 2 [49, 50]. Notably, low doses (0.1 and 0.25 mM) of or acute exposure (from 1 h to 4 h) to H_2_O_2_ increased the Sp1-mediated expression of ZNF32, whereas high doses of H_2_O_2_ (0.5 mM for 24 h) repressed ZNF32 transcription by inhibiting the binding activity of Sp1 (Supplementary Figure S1). This phenomenon could be explained by the kinetics of Sp1 activation: Sp1-DNA binding is an “early” and protective response to oxidative stress that is inhibited when cells become irreversibly “committed” to the apoptotic or senescence pathway [51–54]. Thus, ZNF32 acts as a downstream target of Sp1 and mediates the protective role of Sp1 under oxidative stress.

**Figure 8:**
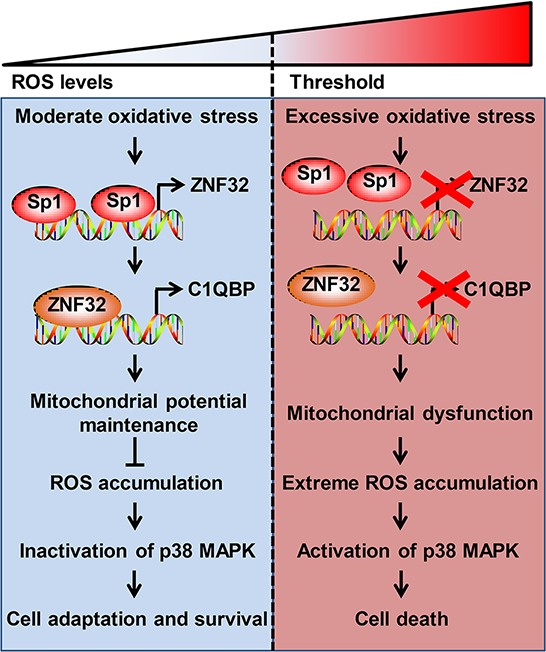
A model for the ZNF32-dependent regulation of ROS homeostasis and apoptosis in response to oxidative stress (Left) Upon exposure to moderate oxidative stress, Sp1 binds to the ZNF32 promoter to increase ZNF32 transcription, which, in turn, increases C1QBP expression, maintains the mitochondrial membrane potential and antioxidant capacity, and inactivates the p38 MAPK pathway, consequently promoting cell survival. (Right) Under excessive oxidative stress, Sp1 is displaced from the ZNF32 promoter, which prevents ZNF32 expression, followed by the suppression of C1QBP transcription, loss of mitochondrial membrane potential, extreme ROS accumulation, activation of p38 MAPK pathway and, ultimately, cell death.

The evidence from our study provides the basis for a model of ZNF32-dependent regulation of apoptosis in response to oxidative stress (Figure 8). Upon the elevation of ROS levels, Sp1 binds to the ZNF32 promoter and augments ZNF32 transcription. Subsequently, C1QBP expression is increased by ZNF32 to maintain mitochondrial membrane potential, activate antioxidant defenses, and consequently reduce the ROS levels to amounts that are not detrimental to cell survival. However, when cells are subjected to excessive oxidative stress above the critical threshold, the expression of ZNF32 is repressed by decreased DNA-binding activity of Sp1, leading to C1QBP downregulation, mitochondrial dysfunction and antioxidant system impairment and ultimately resulting in extreme ROS accumulation, p38 MAPK pathway activation, and cell death. As growing cancer cells *in vivo* are typically subjected to mild oxidative stress, including hypoxia and altered metabolism, ZNF32 performs an important function in the protection of cells from oxidative stress-induced apoptosis, and this function enables cell survival and proliferation. Moreover, increased expression of ZNF32 was significantly associated with poor differentiation in clinical HCC samples, and these results suggest that ZNF32 contributes to the malignant progression of HCC. With the development of tumors, insufficient vascular perfusion causes injurious conditions, such as excessively low pH and nutrient deprivation, and serious oxidative stress in the local tumor microenvironment. These events may result in the suppression of ZNF32 transcription, leading to the impairment of the antioxidant system and, ultimately, cell death. Consistently, we showed that low expression of ZNF32 was significantly associated with the necrosis of tumor areas in clinical HCC samples. Additional evidence indicates that the pathways underlying stress adaptations may represent the most critical weak point in most tumors. Therefore, molecules that mediate such adaptations, rather than oncogenes and tumor suppressors, could be the next important targets for future anticancer drug discovery studies [17, 55, 56]. ZNF32 increases the threshold for oxidative stress-induced cell death. This finding suggests that ZNF32 or one of its target genes could serve as a therapeutic target for cancer treatment. For HCC patients with high ZNF32 expression, ZNF32 inhibition combined with agents that induce oxidative stress represent an efficient strategy for killing cancer cells. Future studies aimed at assessing which transcription factor partners with ZNF32 to control mitochondrial metabolism, the antioxidant system and the tumor microenvironment will help us to more precisely elucidate the role of ZNF32 in tumor progression and therapy.

In conclusion, our study delineates the molecular mechanisms underlying the ZNF32-dependent regulation of apoptosis in response to oxidative stress. The Sp1-mediated transcriptional control of ZNF32 is strongly associated with cell fate in response to the different levels of ROS. The expression of ZNF32 protects against oxidative stress-induced apoptosis via enhanced C1QBP transcription, which maintains mitochondrial membrane potential, followed by inactivation of the p38 MAPK pathway. In contrast, depletion of ZNF32 increases the oxidative damage caused by pro-oxidant agents and enhances tumor suppression both *in vitro* and *in vivo*. Taken together, our data suggest a novel mechanism by which the Sp1-ZNF32-C1QBP axis resists oxidative stress, and we propose that ZNF32 may serve as a therapeutic target to enhance the sensitivity of cancer to pro-oxidant agents.

## MATERIALS AND METHODS

### Cell culture and reagents

HEK293 human embryonic kidney cells and Huh7 and HepG2 HCC cells were maintained in Dulbecco's modified Eagle's medium (DMEM) supplemented with 10% fetal bovine serum, 100 units/ml penicillin, and 100 mg/ml streptomycin. Cells were sustained at 37°C in an atmosphere of 95% humidified air and 5% CO_2_.

The following reagents were used: H_2_O_2_ (323381, Sigma), PL (SML0221, Sigma), SB203580 (S8307, Sigma), NAC (A9165, Sigma), BHA (B1253, Sigma), thiourea (T8656, Sigma), urea (U6504, Sigma), dithiothreitol (D9779, Sigma), protease inhibitor cocktail (P8340, Sigma), TurboFect transfection reagent (R0531, Thermo), ampholyte (#163–1112, BIO-RAD), and CHAPS (#161–0460, BIO-RAD).

### Transient transfection of siRNA and generation of stable cell lines displaying ZNF32 overexpression or knockdown

Specific siRNA targeting C1QBP was synthesized by GenePharma Company (Shanghai, China); the sense sequences were as follows: siRNA-C1QBP, 5′-GGU UGA AGA ACA GGA GCC U-3′; siRNA-NC (negative control), 5′-UUC UCC GAA CGU GUC ACG U-3′. HepG2 cells were seeded in a six-well plate, and 100 pmol of siRNA-C1QBP was transfected into HepG2 cells using TurboFect transfection reagent.

The ZNF32 lentiviral expression vector was constructed by inserting expanded ZNF32 cDNA (NM_006973.2) fragments into a lentiviral shuttle vector (Lv-6). Knockdown of ZNF32 was accomplished using specific shRNA targeting ZNF32. The shRNA sequences were as follows: shRNA-ZNF32, 5′-GAA TGT AGC GTT CTT CAA TGT-3′; shRNA-NC, 5′-TTC TCC GAA CGT GTC AGG T-3′. The shRNA template was cloned into a lentivirus shuttle vector (LV-2: pGLVU6/Puro). The packing and purification of the recombinant lentiviral vector were performed by GenePharma Company (Shanghai, China). Huh7 and HepG2 cells were infected with the recombinant lentiviral vectors at a multiplicity of infection of 100, and the culture supernatant containing lentivirus was removed after 24 h. Cells were selected using 50 μg/ml puromycin.

### Quantitative real-time polymerase chain reaction (qRT-PCR)

To validate the ZNF32-dependent expression of the 10 genes selected for 2-DE analysis, qRT-PCR was performed with SYBR Green Master Mix (TAKARA) using an iCycler iQTM Multicolor Real-Time Detection System (BIO-RAD). The following primers were used: ALDOA, 5′-TGA CAT CGC TCA CCG CAT CG-3′ and 5′-ATC ATC CGC CTT CTG GTA GAG TGT C-3′; C1QBP, 5′-TCA ACA TTA ACA ACA GCA TCC CAC C-3′ and 5′-AGT GCC TTC TTG CCA TCA TCA TTC-3′; GAPDH, 5′-ACC ACA GTC CAT GCC ATC AC-3′ and 5′-TCC ACC ACC CTG TTG CTG TA-3′; HSP90AA1, 5′-TGC CCT TCT ATT TGT CCC ACG-3′ and 5′-GCA AGA TCC TCC GAG TCT ACC AC-3′; HSP90AB1, 5′-TCA GAA GGA TGA CAG CGG TAA GG-3′ and 5′-GAG CCC GAC GAG GAA TAA ATA GC-3′; HSPB1, 5′-TCA AGA CCA AGG ATG GCG TGG T-3′ and 5′-AGG GGA CAG GGA GGA GGA AAC TT-3′; PDIA3, 5′-CAG CAA CTT GAG GGA TAA CTA CCG-3′ and 5′-GTC TTC TGT CAT GTG AGG GCA GAT A-3′; PGAM1, 5′-CGA ATG GAA CCT GGA GAA CCG-3′ and 5′-GTC AAA CTC ATA GCC AGC ATC TCG T-3′; PGK1, 5′-TTC TGA GGC TGT CAC TCG GGC T-3′ and 5′-CAT GGC TGA CTT TAT CCT CCG TGT T-3′; PSME3, 5′-CCA CAA CCC CTG TCA CAT ACC TTT C-3′ and 5′-TCT CTC TGC TCT CCA CCA ACT CAC C-3′; TUBB, 5′-CAC AGG TGG CAA ATA TGT TCC TCG-3′ and 5′-GCC TTT GGC CCA GTT GTT ACC T-3′; and ZNF32, 5′-AGA ATG TAG CGT TCT TCA ATG TG-3′ and 5′-CCT GTA GTG TCT TCG AAT CTG G-3′. The relative expression levels were determined using Gene Expression Macro Version 1.1 software (BIO-RAD).

### Western blot analysis

The cells were collected and washed with PBS three times and then lysed with lysis buffer (50 mM Tris–HCl, 150 mM NaCl, 1 mM EDTA, 50 mM NaF, 30 mM Na_4_P_2_O_7_, 1 mM phenylmethylsulfonyl fluoride, and 2 μg/ml aprotinin) for 30 min on ice. After the determination of the protein content using the Bio-Rad Protein Assay (BIO-RAD), equal amounts of extracted protein were loaded, separated via 12% SDS-PAGE and transferred to a polyvinylidene difluoride membrane (Millipore). After blocking with TBS (10 mM Tris–HCl, pH 8.0, and 150 mM NaCl) containing 5% skimmed milk for 1 h at 37°C, the membrane was incubated in a primary antibody at 4°C overnight. Immunoblotting was performed using antibodies against p38 MAPK (sc-535, Santa Cruz Biotechnology), phospho-p38 MAPK (#4511, Cell Signaling Technology), MAPKAPK-2 (sc-7871, Santa Cruz Biotechnology), phospho-MAPKAPK-2 (#3007, Cell Signaling Technology), cleaved caspase-3 (#9664, Cell Signaling Technology), cleaved PARP (#5625, Cell signaling Technology), C1QBP (#6502, Cell Signaling Technology), PGK1 (sc-17943, Santa Cruz Biotechnology), HSPB1 (#2402, Cell Signaling Technology), Sp1 (sc-59X, Santa Cruz Biotechnology), FLAG (F7425, Sigma), 8-OHdG (ab26842, Abcam). An antibody against β-actin (sc-47778, Santa Cruz Biotechnology) was used as a loading control. The rabbit polyclonal anti-ZNF32 antibody was generated by immunizing rabbits with a recombinant peptide of human ZNF32 [57]. After incubation in a primary antibody, the membrane was washed in TBST (TBS containing 0.1% Tween 20) three times and then incubated in a horseradish peroxidase (HRP)-conjugated goat anti-rabbit/anti-mouse antibody (Santa Cruz Biotechnology) for 1 h at 37°C. After three washes with TBST, the membrane was developed using Immobilon™ Western Chemiluminescent HRP Substrate (Millipore).

### Generation of ZNF32 promoter constructs and site-directed mutagenesis

Different sections of the 5′-flanking region of the human ZNF32 gene were generated by PCR using the following forward primers:

ZNF32 (−1443/+66), 5′-GTCAGGTACCTTCAC GGCAGGATCTGAAAC-3′;

ZNF32 (−1139/+66), 5′-ATTGTCGGTACCTCCC TAGGAGCACGG-3′;

ZNF32 (−884/+66), 5′-ATTAGGGTACCACGAAA GAGGATGGACAGACAC-3′;

ZNF32 (−509/+66), 5′-CTTCGGTACCTACGTG TTCAAAAGCAACCAT-3′;

ZNF32 (−386/+66), 5′-AGCCGGTACCTCAACA CATGACCTCGAAG-3′; and

ZNF32 (−178/+66), 5′-ACCGGGTACCGTCTCCG GATTACCAG-3′. The following reverse primer was used for the generation of all ZNF32 promoter reporter constructs: 5′-GCAG*AAGCTT*GTGCCCGCAGACAA-3′. *Kpn*I restriction sites are underlined, and the *Hind*III restriction site is in italics. All PCR products were cloned with *Kpn*I/*Hind*III into the luciferase-based vector pGL3-basic (Promega). Mutations in the Sp1 binding sites were generated using the Fast Mutagenesis System (Transgen Biotech, China). The following primers were used, with mutations in italics:

Sp1-Mut-1 primer, 5′-GGC CTT TGC TGT GTG C*TT T*CC TCC ACC CCT-3′ (forward), 5′-*AAA* GCA CAC AGC AAA GGC CAA GGC TCA GAG TCC-3′ (reverse);

Sp1-Mut-2 primer, 5′-AGA CCC ACG GCC CGG *TTT* GCG GGC TCG CG-3′ (forward), 5′-*AAA* CCG GGC CGT GGG TCT CGG CGG CCC C-3′ (reverse).

### Dual luciferase reporter assay

For cotransfection, HEK293 cells were transfected with 0.25 μg of a ZNF32 promoter reporter construct and 0.05 μg of pRL-TK with or without 0.75 μg of pCGN-Sp1 using 2 μl of TurboFect per well in 48-well plates. Twenty-four hours post-transfection, the cells were rinsed twice with PBS and incubated in medium containing 0.5 mM H_2_O_2_ for an additional 24 hours. The cells were then lysed in Passive Lysis Buffer, and luciferase activity was measured in the cell lysates. The dual-luciferase reporter assay was performed according to the manufacturer's instructions (Promega) using a Multi-Mode Microplate Reader (Synergy 2, BioTek).

### EMSAs

Nuclear protein extracts were prepared using NE-PER Nuclear and Cytoplasmic Extraction Reagents (Thermo) according to the manufacturer's instructions. Approximately 10 μg of nuclear extract was incubated in 5′-end biotin-labeled double-stranded oligonucleotides. The binding reaction was performed for 20 minutes at room temperature using the LightShift Chemiluminescent EMSA Kit (Thermo). To resolve protein-DNA complexes, the reaction mix was separated on a 5% nondenaturing gel in 0.5 × Tris-borate EDTA. The gel was transferred to a positively charged nylon membrane (Millipore), and the membrane was developed using the Chemiluminescent Nucleic Acid Detection Module (Thermo) according to the manufacturer's instructions. For competition assays, nuclear extracts were incubated in a 200-fold molar excess of unlabeled double-stranded competitor oligonucleotides.

### ChIP assays

ChIP assays were performed using the EZ-Magna ChIP™ A kit (Millipore) according to the manufacturer's protocol. Aliquots of chromatin immunoprecipitation lysates were diluted and incubated in the indicated antibody and protein A magnetic beads at 4°C for 2 h. The beads were separated using a magnetic separator and sequentially washed with low-salt buffer, high-salt buffer, LiCl Immune Complex Wash Buffer and TE Buffer. After de-cross-linking and proteinase K digestion, the DNA was cleaned using a spin filter. PCR was performed in the exponential linear zone of amplification using the following primers:

ZNF32 gene promoters, ^−1409^ACA GCC GGT CTT GAC CTT AAC^−1388^ (forward) and ^−1213^GGG GTG TTG CAC AGC TAA GTC^−1193^ (reverse); ^−185^GCT GTG AGT CTC CGG ATT ACC AG^−163^ (forward) and ^+75^CGG CCT CAC TCA CCG CA^+91^ (reverse); C1QBP gene promoters, ^−1133^CAC AAG CAT GAG TTC CGA GCC^−1113^ (forward) and ^−966^GTT GGG ATT GAA TTG GAG GAC AC^−944^ (reverse).

### Oxidative damage to lipids

MDA, a secondary product of lipid peroxidation, was measured using the MDA assay kit (Jiancheng Bioengineering Institute, Nanjing, China). Cell culture supernatants were used because it had been shown that MDA is almost completely released into the culture medium within 1 h after production [58]. Briefly, the hydrolysate of lipoperoxides was reacted with thiobarbituric acid (TBA) in a 95°C water bath for 40 min to yield a red MDA-TBA adduct. Supernatants containing the MDA-TBA adduct were measured at 532 nm using a microplate spectrophotometer (uQuant MQX200, BioTek). The concentration of MDA was calculated according to the manufacturer's instructions.

### Measurement of intracellular ROS

To assess the generation of intracellular ROS, cells were washed with DMEM twice and then incubated in 5 μM DCFH-DA (Jiancheng Bioengineering Institute, Nanjing, China) at 37°C for 30 min. The cells were washed with PBS three times and lysed in lysis buffer after incubation. Then, the fluorescence intensity was measured using a Fluoroskan Ascent FL microplate fluorometer (Thermo) with excitation and emission settings of 485 nm and 530 nm, respectively.

### Measurement of catalase activity

Catalase activity was determined using assay kits for catalase according to the manufacturer's instructions (Jiancheng Bioengineering Institute, Nanjing, China). The results were presented as specific activity, which was calculated as the total activity per sample divided by the total amount of protein per sample.

### Flow cytometric analysis

Apoptotic cells were quantified via Annexin V-FITC and PI double-staining using a staining kit from KeyGEN Biotech (Nanjing, China). Briefly, cells were trypsinized with trypsin, washed twice with PBS, resuspended in 200 μl of binding buffer containing 2 μl of Annexin V-FITC and 2 μl of PI and incubated at room temperature for 15 min. Then, the cell mixture was immediately analyzed using a flow cytometer (FACSAria, Becton Dickinson).

The mitochondrial membrane potential was measured using a mitochondria-specific dual fluorescence probe, JC-1 (Beyotime), as described previously [59]. Briefly, cells were washed twice with PBS and loaded with JC-1-containing solution for 30 min at 37°C. JC-1 fluorescence was quantified by flow cytometry, in which red JC-1 aggregates were gated in the FL2 channel and green JC-1 monomers in the FL1 channel. Analysis of the multivariate data was performed using FlowJo software.

### Tumor xenograft experiments

For these experiments, 6-week-old BALB/c nude mice were subcutaneously inoculated in the flank region with either 5 × 10^6^ shZNF32-HepG2 cells or 5 × 10^6^ shNC-HepG2 cells. When the diameter of HepG2 tumors reached approximately 3 mm, the tumor-bearing mice were randomly selected for treatment with DMSO control or PL (2.5 mg/kg/day) via intraperitoneal injection for 15 days. The tumor size was measured every 3 days until the mice were sacrificed. The tumor volume was calculated using the following formula: tumor volume (mm^3^) = π/6 × length × width^2^. The tumors were weighed after the mice were sacrificed. All animals received humane care according to the Institutional Animal Care and Treatment Committee of Sichuan University.

### TUNEL assay

The *in situ* cell death assay was performed using a TUNEL kit (Promega) in accordance with the manufacturer's instructions. The slides were observed under a fluorescence microscope (Olympus Optical Co., Hamburg, Germany), and a nucleus displaying bright green fluorescent staining was recorded as a TUNEL-positive cell. The number of TUNEL-positive cells was counted in 5 independent randomly selected fields. These values were expressed as the percentage of positive cells relative to the total number of cells.

### Clinical specimens

All HCC and corresponding adjacent normal tissues were obtained from patients who underwent surgical resection at West China Hospital (Chengdu, China). All paired samples were immediately frozen in liquid nitrogen. Ethical approval was obtained from the Institutional Ethics Committee of West China Hospital. The fibrosis score, histodifferentiation and TNM stage were characterized according to the AJCC classification system [60]. The clinical pathological information for these patients is summarized in Table 2.

### 2-DE and MS/MS analysis

2-DE-based proteomic analysis was performed as described previously [61]. Briefly, the indicated cells were dissolved in lysis buffer (7 M urea, 2 M thiourea, 4% CHAPS, 100 mM dithiothreitol, and 0.2% pH 3–10 ampholyte) in the presence of protease inhibitor. Samples were loaded on IPG strips (17 cm, pH3–10NL, BIO-RAD) using a passive rehydration method and then subjected to isoelectric focusing (BIO-RAD). Separation in the second dimension was performed using 12% SDS-PAGE. The gels were stained with Coomassie Brilliant Blue R-250 (BIO-RAD). In-gel protein digestion was performed using MS-grade trypsin (Trypsin Gold, Promega) according to the manufacturer's instructions. Gel spots were destained with 100 mM NH_4_HCO_3_/50% acetonitrile (ACN) and then dehydrated with 100% ACN. The gels were then incubated in trypsin, followed by double extraction with 50% ACN/5% trifluoroacetic acid. The peptide extracts were dried in a speed-VAC concentrator (Thermo) and subjected to mass spectrometric analysis using a Q-TOF mass spectrometer (Micromass, UK) fitted with an ESI source.

### Statistical analysis

All data are expressed as the means ± SEM. To evaluate the significance of the differences between two groups, the means were compared using Student's t test. Multiple-group comparisons were performed via one-way analysis of variance. The data were analyzed using SPSS statistical software version 22.0. *p* < 0.05 was considered to be significant.

## SUPPLEMENTARY FIGURES



## Abbreviations

8-OHdG: 8-hydroxydeoxyguanosine
BHA: butylated hydroxyanisole
C1QBP: complement 1q-binding protein
HCC: hepatocellular carcinoma
ChIP: chromatin immunoprecipitation
EMSA: electrophoretic mobility shift assay
KLF: Krüppel-like family of transcription factors
MAPK: mitogen-activated protein kinase
MDA: malondialdehyde
mPTP: mitochondrial permeability transition pore
mTERT: mouse telomerase reverse transcriptase
NAC: N-acetyl-L-cysteine
PARP: poly(ADP-ribose) polymerase
PL: piperlongumine
qRT-PCR: quantitative real-time polymerase chain reaction
ROS: reactive oxygen species
siRNA: small interfering RNA
shRNA: short hairpin RNA
Sp1: Sp1 transcription factor
ZNF: zinc finger protein
