# Supplementary material for: ZNF32 protects against oxidative stress-induced apoptosis by modulating C1QBP transcription
Source: Oncotarget. 2015 Oct 16;6(35):38107–26. doi: 10.18632/oncotarget.5646 (PMC4741987; doi:10.18632/oncotarget.5646)
Supplement: Supplementary file 1 [file oncotarget-06-38107-s001.pdf]

SUPPLEMENTARY FIGURES

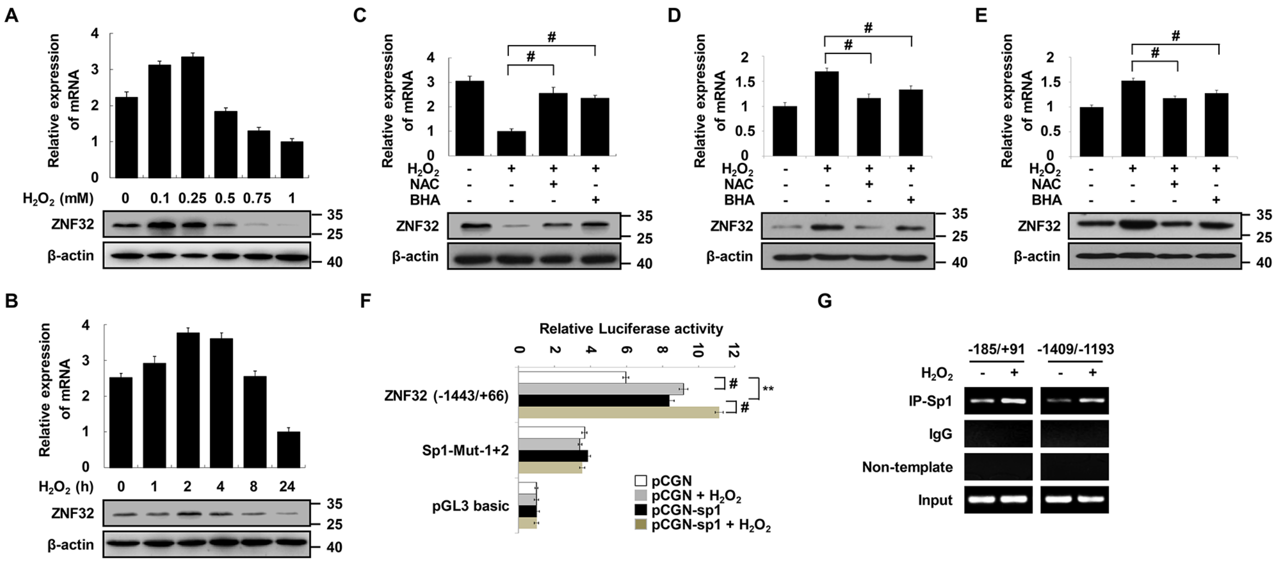

Supplementary Figure S1: Oxidative stress regulates the expression of ZNF32. A. HepG2 cells were stimulated with increasing concentrations of H_2O_2 for 24 h and then subjected to qRT-PCR and immunoblot analysis. **B.** HepG2 cells were exposed to 0.5 mM H_2O_2 for the indicated periods and then subjected to qRT-PCR and immunoblot analysis. **C.** HepG2 cells were pretreated with 1 mM NAC (an antioxidant) or 100 µM BHA for 1 h, treated with 0.5 mM H_2O_2 for 24 h, and then subjected to qRT-PCR and immunoblot analysis. **D.** HepG2 cells were pretreated with NAC or BHA for 1 h, treated with 0.1 mM H_2O_2 for 24 h, and then subjected to qRT-PCR and immunoblot analysis. **E.** HepG2 cells were pretreated with NAC or BHA for 1 h, treated with 0.75 mM H_2O_2 for 1 h, and then subjected to qRT-PCR and immunoblot analysis. **F.** HEK293 cells were transiently transfected with the indicated constructs, treated with 0.1 mM H_2O_2 for 24 h and then analyzed using a dual luciferase reporter assay. **G.** DNA fragments from HEK293 cells treated as in (F) were immunoprecipitated with Sp1-specific antibodies and analyzed via RT-PCR using the indicated primers. The data are presented as the mean values ± SEM. Each experiment was performed at least in triplicate, producing consistent results. **p* < 0.05, ***p* < 0.01, #*p* < 0.001.

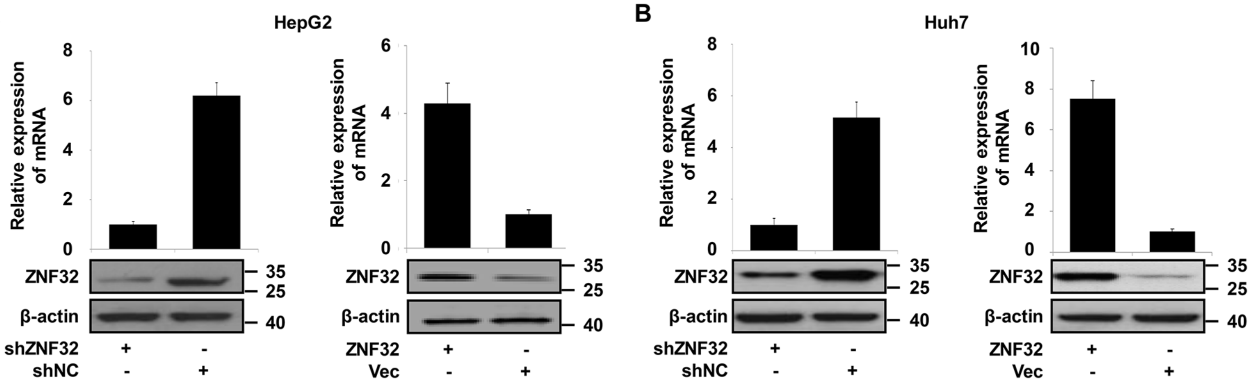

Supplementary Figure S2: Identification of stable overexpression or knockdown of ZNF32 in HepG2 and Huh7 cells. HepG2 **A.** and Huh7 **B.** cells were infected with the indicated lentiviral constructs, selected with puromycin and then subjected to immunoblot analysis using the indicated antibodies.

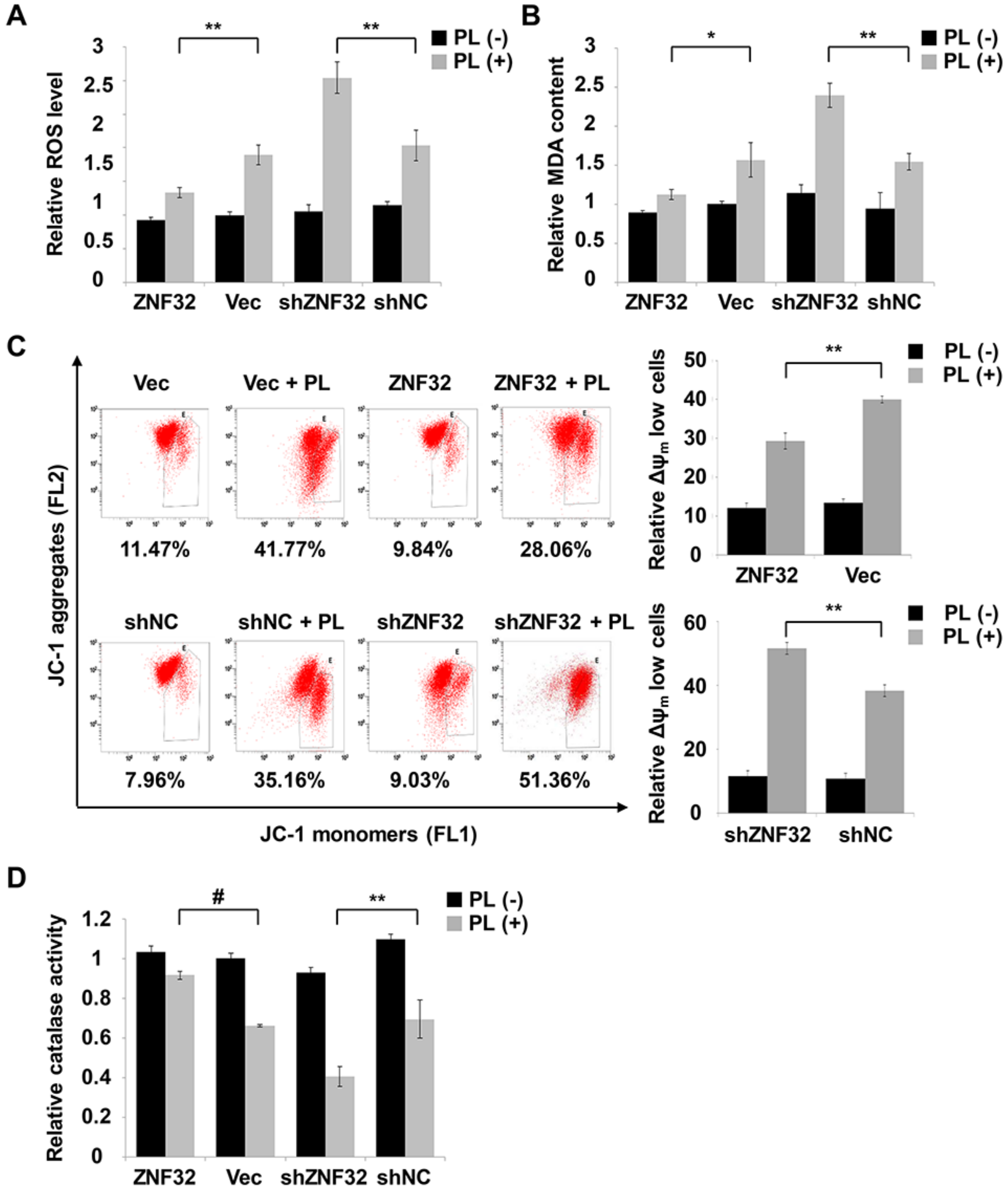

Supplementary Figure S3: ZNF32 protects against oxidative stress by maintaining the mitochondrial membrane potential and antioxidant capacity. A. The indicated HepG2 cells were incubated in 10 µM PL for 24 h and then stained with DCFH-DA, and DCF fluorescence intensity was then determined using a fluorescence microplate reader. **B.** The indicated HepG2 cells were treated as in (A) and then assessed for the lipid peroxidation level using an MDA assay. **C.** The indicated HepG2 cells were treated as in (A) and then subjected to measurement of the mitochondrial membrane potential using a JC-1 assay. **D.** The indicated HepG2 cells treated as in (A) were subjected to catalase activity analysis to determine the intracellular antioxidant capacity. The data are presented as the mean values ± SEM. Each experiment was performed at least in triplicate, producing consistent results. *$p < 0.05$, **$p < 0.01$.

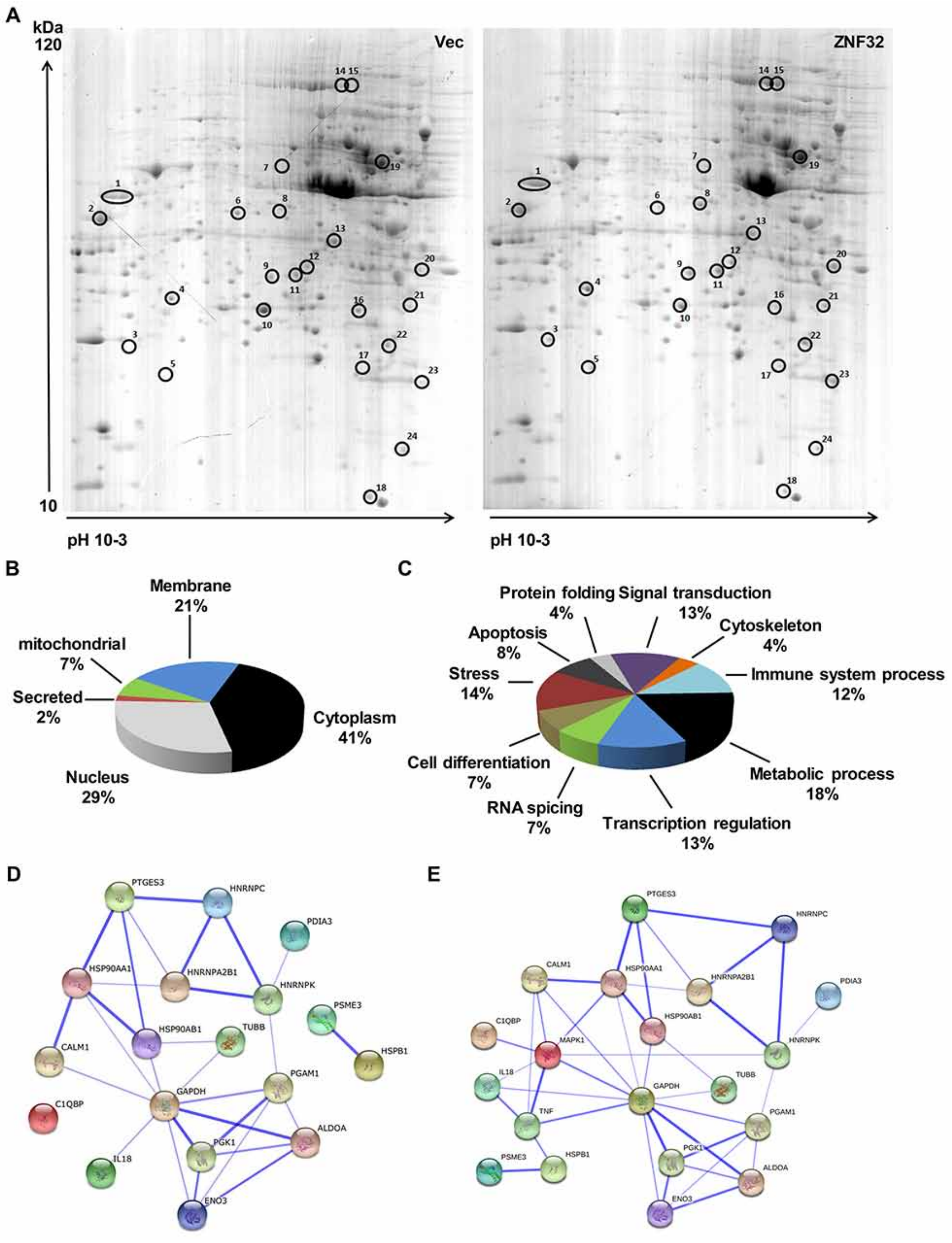

Supplementary Figure S4: Proteomic analysis of ZNF32-overexpressing HepG2 cells. A. Representative two-dimensional gel images of HepG2 cells stably expressing ZNF32 or a null vector. Total protein extracts were separated on pH 3–10 nonlinear IPG strips in the first dimension and by 12% SDS-PAGE in the second dimension. Then, the proteins were visualized by Coomassie Brilliant Blue staining. Gel spots were selected for *in situ* gel digestion and analyzed using ESI-Q-TOF tandem mass spectrometry. A total of 24 differentially expressed spots (15 upregulated and 9 downregulated in ZNF32-overexpressing HepG2 cells) were identified (as numbered). Details for each numbered spot are reported in Table I. **B.** The identified proteins were categorized as cytoplasmic (41%), nuclear (29%), mitochondrial (7%), cell membrane (21%), or secreted (2%) proteins. **C.** These identified proteins were classified into 10 groups, including metabolic process (18%), stress (14%), signal transduction (13%), apoptosis (8%), and cell differentiation (7%). **D.** and **E.** In silico protein interaction analysis. The identified proteins involved in oxidative stress and apoptosis were analyzed for protein interactions using the web based software tool STRING either without (D) or with (E) the addition of MAPK1 and TNFα, which are critical effectors of ROS-associated signaling.

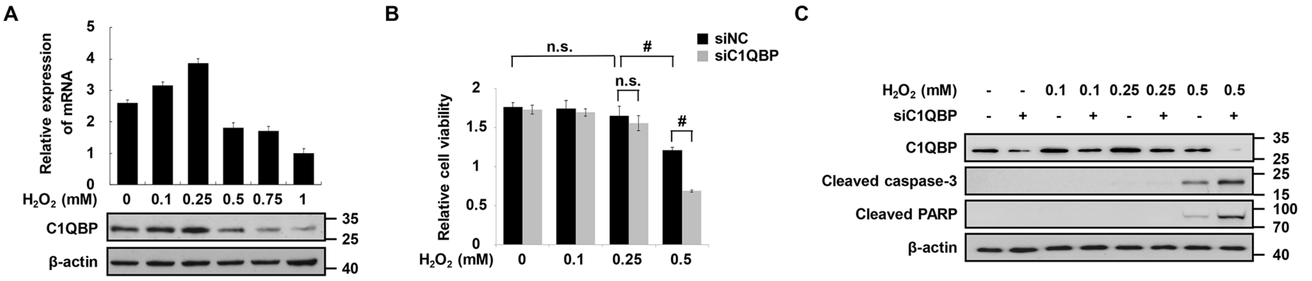

Supplementary Figure S5: The protective effects of C1QBP to resist oxidative stress. A. HepG2 cells were treated with increasing concentrations of H_2O_2 for 24 h and then subjected to qRT-PCR and immunoblot analysis. **B.** HepG2 cells were transfected with siC1QBP, treated with indicated concentrations of H_2O_2 for 24 h and then analyzed for cell viability. **C.** HepG2 cells were transfected and treated as in (B) and then subjected to immunoblot analysis using the indicated antibodies. The data are presented as the mean values ± SEM. Each experiment was performed at least in triplicate, producing consistent results. #$p < 0.001$.

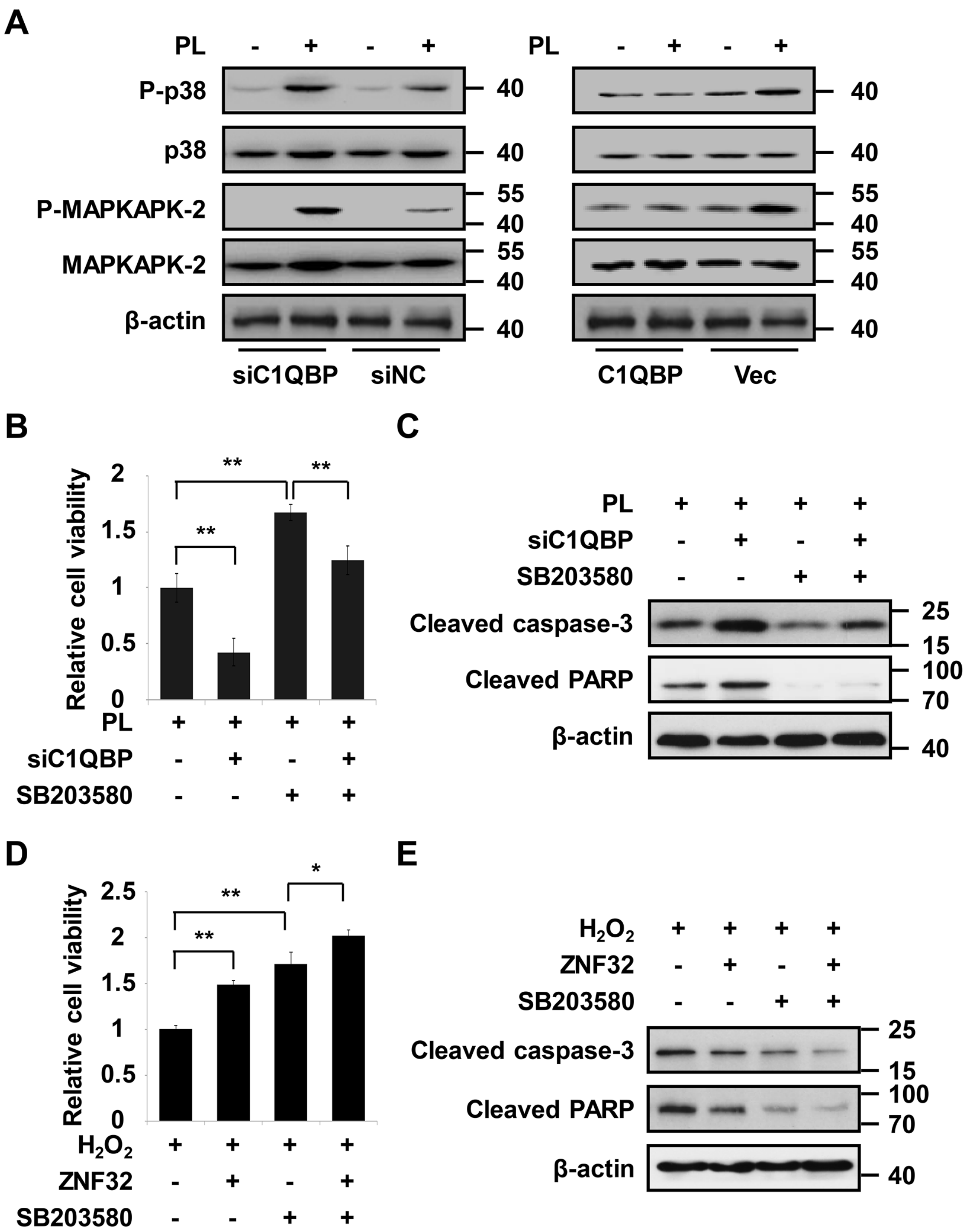

Supplementary Figure S6: C1QBP knockdown facilitates the activation of p38 MAPK upon oxidative stress. A. HepG2 cells were transfected with a C1QBP expression plasmid or siC1QBP, treated with 10 µM PL for 24 h and then subjected to immunoblot analysis using the indicated antibodies. **B.** HepG2 cells were transfected with siC1QBP, pretreated with SB203580 for 1 h, treated with 10 µM PL for 24 h and then analyzed for cell viability. **C.** HepG2 cells were transfected and treated as in (B) and then subjected to immunoblot analysis using the indicated antibodies. **D.** The indicated HepG2 cells were pretreated with SB203580 for 1 h, treated with 0.5 mM H_2O_2 for 24 h and then analyzed for cell viability. **E.** The indicated HepG2 cells were treated as in (D) and subjected to immunoblot analysis using the indicated antibodies. The data are presented as the mean values ± SEM. Each experiment was performed at least in triplicate, producing consistent results. *$p < 0.05$, **$p < 0.01$, #$p < 0.001$.
